# Lung cavitation in COVID-19: co-infection complication or rare evolution?

**DOI:** 10.31744/einstein_journal/2020AI5822

**Published:** 2020-07-17

**Authors:** Lucas Tadashi Wada Amaral, Gabriel Laverdi Beraldo, Vanessa Mizubuti Brito, Marcela Emer Egypto Rosa, Marina Justi Rosa de Matos, Eduardo Kaiser Ururahy Nunes Fonseca, Patrícia Yokoo, Murilo Marques Almeida Silva, Gustavo Borges da Silva Teles, Hamilton Shoji, Rodrigo Bastos Duarte Passos, Rodrigo Caruso Chate, Gilberto Szarf

**Affiliations:** 1 Hospital Israelita Albert Einstein São PauloSP Brazil Hospital Israelita Albert Einstein, São Paulo, SP, Brazil.

**Figure 1:**
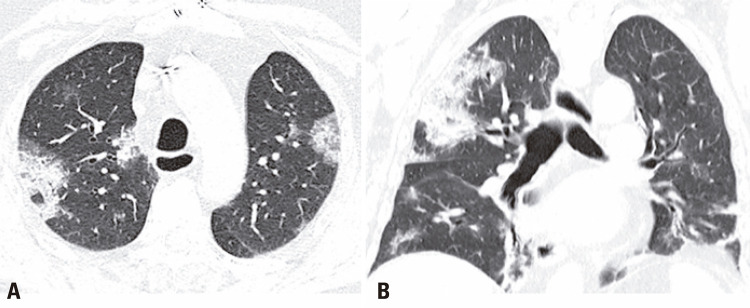
Axial and coronal chest computed tomography shows multiple and bilateral ground-glass opacities with areas of consolidation and septal thickening, typical findings of COVID-19

**Figure 2:**
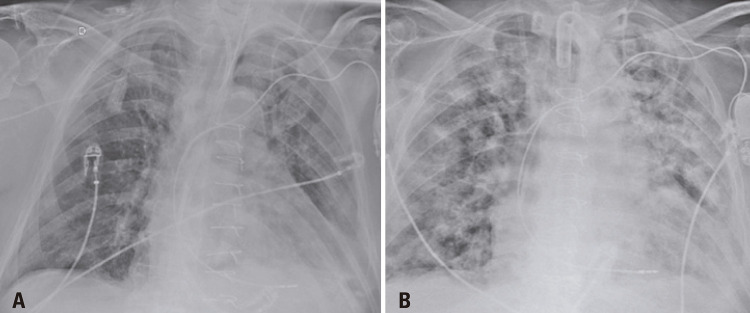
Bedside anteroposterior chest X-ray images demonstrate the radiographic evolution of the nosocomial infection with multiple areas of consolidation in both lungs

**Figure 3:**
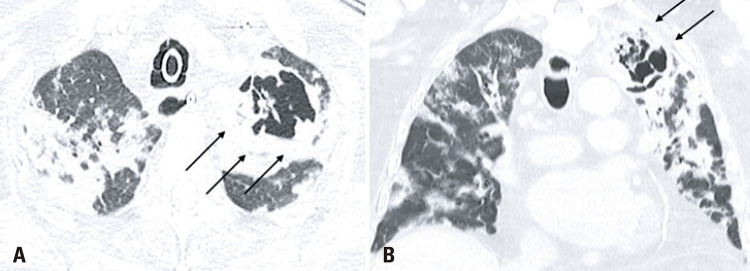
Axial and coronal chest computed tomography images show diffuse areas of consolidation, ground-glass opacities and a cavitating lesion in the superior lobe of the left lung (black arrows)

An 86-years-old male patient was admitted to the emergency department with a 1-day history of fever, dyspnea, and cough. After an assessment, there were no criteria that justified the patient’s hospitalization, therefore, he was dismissed and instructed to continue treatment at home. One week after the onset of symptoms, the patient returned with worsening dyspnea and persistent fever, which prompted his hospitalization. A chest computed tomography (CT) demonstrated typical coronavirus disease (COVID-19) findings^(1,2)^ (Figure 1), and the real-time polymerase chain reaction (rt-PCR) test confirmed the diagnosis. After 13 days of hospitalization, the patient experienced clinical worsening, and nosocomial pneumonia caused by *Enterococcus faecalis* was diagnosed (Figure 2). The following CT 10 days after the diagnosis of the nosocomial infection showed multiple bilateral areas of ground-glass opacities, accompanied by septal thickening and consolidation areas, possibly related to COVID-19. There was also an excavated new lesion in the left upper lobe (Figure 3). A new rt-PCR test was still positive for COVID-19 on the same day of this last chest CT scan.

Recent studies have demonstrated that pulmonary co-infection caused by other agents is not uncommon in the COVID-19 context.^(3,4)^ Complications such as necrotizing pneumonia and subsequently excavated lung lesions become possible, and their imaging presentation is considered an atypical finding in COVID-19.^(5)^

The persistent positive COVID-19 rt-PCR seen in this case did not allow us to differentiate whether findings of pulmonary excavation were determined by the bacterial co-infection or were related to the COVID-19 infection alone.

Even though many clinical and imaging aspects of COVID-19 have already been elucidated, some cases of the novel coronavirus continue to surprise the medical team. Further studies are still needed to develop a better understanding of this new virus.
