# Supplementary material for: Lung cavitation in COVID-19: co-infection complication or rare evolution?
Source: Einstein (Sao Paulo). 2020 Jul 17;18:eAI5822. doi: 10.31744/einstein_journal/2020AI5822 (PMC7367597; doi:10.31744/einstein_journal/2020AI5822)
Supplement: Translation

# Translation: Escavação pulmonar em paciente com COVID-19: complicação de coinfecção ou evolução rara?

**Authors:** Lucas Tadashi Wada Amaral, Gabriel Laverdi Beraldo, Vanessa Mizubuti Brito, Marcela Emer Egypto Rosa, Marina Justi Rosa de Matos, Eduardo Kaiser Ururahy Nunes Fonseca, Patrícia Yokoo, Murilo Marques Almeida Silva, Gustavo Borges da Silva Teles, Hamilton Shoji, Rodrigo Bastos Duarte Passos, Rodrigo Caruso Chate, Gilberto Szarf

**Affiliations:** 1 Brasil Hospital Israelita Albert Einstein, São Paulo, SP, Brasil.

Paciente do sexo masculino com 86 anos de idade, foi admitido no nosso serviço de emergência com história de 1 dia de febre, dispneia e tosse. Após avaliação, não houve critérios que justificassem sua internação, sendo liberado com orientações e tratamento domiciliar. Uma semana após o aparecimento dos sintomas, o paciente retornou ao serviço demostrando piora da dispneia e persistência da febre, o que motivou sua internação. A tomografia computadoriza (TC) do tórax demonstrou achados típicos de infecção pelo coronavírus (COVID-19)^(1,2)^ (Figura 1), e o resultado do teste de reação em cadeia da polimerase (PCR) em tempo real confirmou o diagnóstico. No décimo terceiro dia de hospitalização, o paciente apresentou degeneração clínica, sendo diagnosticado com pneumonia nosocomial causada por *Enterococcus faecalis* (Figura 2). Nova TC 10 dias após o diagnóstico da infecção hospitalar mostrou múltiplas opacidades em vidro fosco, juntamente de espessamento septal e áreas de consolidação, achados possivelmente ainda relacionados à COVID-19. Observou-se também uma lesão escavada no lobo superior esquerdo (Figura 3), achado novo em relação à última tomografia. Novo teste de PCR demostrou infecção ainda positiva para COVID-19 no mesmo dia da última TC.

**Figura 1:** Tomografia computadorizada do tórax em plano axial e coronal ilustrando opacidades em vidro fosco bilaterais e multifocais, associadas a espessamento septal e fino reticulado, além de focos de consolidação, achados típicos da COVID-19

**Figura 2:** Radiografia do tórax obtida no leito demostrando evolução de infecção hospitalar com múltiplas áreas de consolidação em ambos os pulmões

**Figura 3:** Imagens da tomografia computadorizada do tórax em plano axial e coronal mostrando áreas de consolidação, opacidades em vidro fosco e uma lesão escavada no lobo superior do pulmão esquerdo (setas)

Estudos recentes têm demostrado que a coinfecção pulmonar causada por outros agentes não é incomum no contexto da COVID-19.^(3,4)^ Complicações como pneumonia necrotizante e subsequente escavação pulmonar tornam-se possíveis, considerando que este achado não é típico para COVID-19^(5)^

A positividade persistente da PCR para COVID-19 observada neste caso não permitiu diferenciar se os achados da escavação pulmonar foram determinados pela coinfecção bacteriana ou se estavam associados somente à infecção pela COVID-19.

Apesar do esclarecimento de muitos aspectos clínicos e de imagem da COVID-19, alguns casos do novo coronavírus surpreendem a equipe médica. Novos estudos são necessários para melhor compreender este novo vírus.
